# Conservation genomics and pollination biology of an endangered, edaphic-endemic, octoploid herb: El Dorado bedstraw (*Galium californicum* subsp. *sierrae*; Rubiaceae)

**DOI:** 10.7717/peerj.10042

**Published:** 2020-10-26

**Authors:** Dylan Burge

**Affiliations:** Department of Ecology and Evolutionary Biology, University of California, Los Angeles, Los Angeles, CA, USA

**Keywords:** Asexual, ddRAD, El Dorado county, Nuclear genome, Pine Hill, Rare

## Abstract

El Dorado bedstraw (*Galium californicum* subsp. *sierrae*) is a federally endangered dioecious, octoploid, perennial herb found only in the Pine Hill region of El Dorado County, CA, USA. Like many species of *Galium*, El Dorado bedstraw is capable of both sexual and asexual reproduction, spreading via stem-layering as well as seeds. El Dorado bedstraw is also dioecious, and thus dependent on pollinators to transfer pollen from male to female stems. The capacity for asexual reproduction has conservation implications for this plant, due to the potential for populations to become dominated by a small number of clones in the absence of recruitment from seeds. No previous work has examined either the population genetics or pollination biology of this plant. Here, double-digest restriction site-associated DNA sequencing was used to develop a genetic dataset for a sample of El Dorado bedstraw (12 individuals from each of seven locations). Genomic data was used to calculate population genetic statistics and quantify the degree to which clonality affects the sampled populations. Visual observation of insect visitors at every sampling location was used to assess the potential for pollen transfer within and among locations. A total of 23 clonal colonies were detected across 82 successfully sequenced stems, consisting of an average of 2.4 individuals (range: 2–6). Significant isolation by distance among locations was detected using a Mantel test. Insect pollinators were from eleven families, consisting mainly of small species with weak flight. It is recommended that clonality and small-scale population differentiation be taken into account in conservation measures.

## Introduction

**Figure 1:**
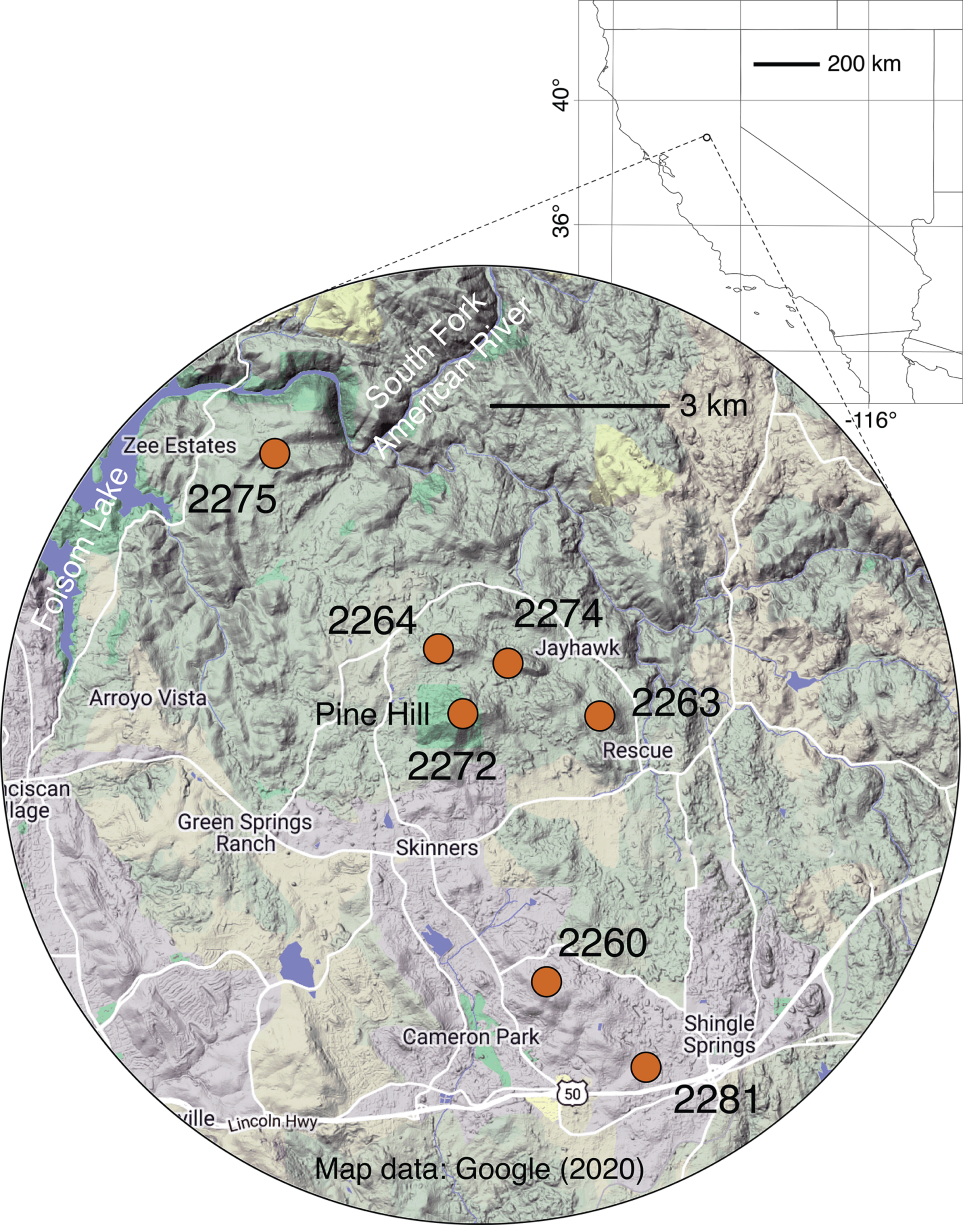
Map of sampling locales. Grid dimensions on the background map are in degrees of latitude and longitude (WGS84 datum). For more information on locales, see Table 1 and Table S1. Background map data © 2020 Google.

*Galium californicum* Hook. and Arn. subsp. *sierrae* Dempster and Stebbins (hereafter referred to by its common name, El Dorado bedstraw) is a federally endangered perennial herb found only in the Pine Hill region of El Dorado County, CA, USA (Fig. 1). El Dorado bedstraw occurs mainly in the understory of forest and chaparral habitat (Soza, 2012). It occurs only on soil derived from gabbro rock of the Pine Hill Formation (Wilson, 1986; Hunter & Horenstein, 1991; USFWS, 2002; Wilson et al., 2009). Because it is restricted to gabbro-derived soil, this taxon is considered an edaphic-endemic (soil-limited) taxon. El Dorado bedstraw is one of five federally threatened or endangered plants that are associated with gabbro-derived soils of the Pine Hill area, a region well known for its elevated botanical diversity and endemism (Wilson, 1986; Hunter & Horenstein, 1991; USFWS, 2002; Wilson et al., 2009). The endangered taxa include Stebbins’ morning-glory (*Calystegia stebbinsii* Brummitt), Pine Hill ceanothus (*Ceanothus roderickii* W. Knight), Pine Hill flannelbush (*Fremontodendron decumbens* R. M. Lloyd) and Layne’s ragwort (*Packera layneae* (Greene) W.A. Weber and Á. Löve).

**Table 1:** Sampling.

Locale name	Code	Sex balance			Latitude	Longitude	Elevation (m)
		♀	♂	Unknown			
Cameron Park	2260	9	3	0	38.6782	−120.9712	475
Tiffany Hill	2263	5	7	0	38.7212	−120.9617	435
Farview	2264	7	5	0	38.7317	−120.9945	480
Pine Hill	2272	10	2	0	38.7214	−120.9895	590
Lazy Knoll	2274	6	6	0	38.7294	−120.9801	480
Salmon Falls	2275	3	9	0	38.7629	−121.0274	286
Many Oaks	2281	9	1	2	38.6643	−120.9498	450

**Note:**

Code, a field collection code for each locale (Fig. 1) used as a short-hand to refer to individual locales; each code corresponds to an herbarium voucher (collector: D. O. Burge) deposited at the UC Davis Center for Plant Diversity. Sex balance, the number of stems of each sex; these are the same stems selected for genotyping; Latitude and longitude are in the WGS 84 datum. Also see Table S1.

El Dorado bedstraw is thought to reproduce vegetatively via branch layering (USFWS, 1996). In this mode of reproduction, each seedling has the ability to form a group of genetically identical individuals (hereafter referred to as a genet) in which all stems (hereafter referred to as ramets) are derived clonally from the same original seed (Harper, 1977). It is not known how frequent sexual versus asexual reproduction is in El Dorado bedstraw. However, because it is dioecious, El Dorado bedstraw depends on pollinators to transfer pollen from male to female flowers. Because most of the close relatives of El Dorado bedstraw are dioecious (Soza & Olmstead, 2010b), there is a stronger potential for interspecific genetic exchange than might be expected among perfect-flowered species capable of selfing. However, the only close relatives known to co-occur with the focal taxon differ in ploidy (Dempster & Stebbins, 1968): El Dorado bedstraw is octoploid (2*n* = 88), *Galium bolanderi* A. Gray is hexaploid (2*n* = 66), and *Galium porrigens* Dempster is diploid (2*n* = 22). These differences in chromosome number imply that genetic exchange is rare, if not entirely absent between El Dorado bedstraw and other native *Galium* found in the Pine Hill area (V. Soza, 2018, personal communication). Furthermore, although the focal taxon is octoploid, it is not known whether it is auto-or alloploid.

El Dorado bedstraw is part of *Galium* sect. *Baccogalium*, a group that is diverse in western North America, and also rich in rare species (Soza & Olmstead, 2010a, 2010b). *Galium californicum* contains a total of seven subspecies, including the nominate race. All of these are endemic to the state of California, and all but El Dorado bedstraw are restricted to the coastal or south-western parts of the state (Soza, 2012). Thus, El Dorado bedstraw occurs far outside the range of all of its putative closest relatives.

Throughout its very small geographic range, El Dorado bedstraw faces threats from urban development and other human activities (e.g., off-highway vehicle use, illegal dumping), which have led to the loss of some populations and fragmentation of remaining ones, especially near the city of Cameron Park (USFWS, 1996; Fig. 1). In addition to the threat posed by development, the dioecious sexual system of the plant, combined with a propensity for asexual reproduction, makes this taxon vulnerable to habitat fragmentation. It is known that in dioecious plants like El Dorado bedstraw, the balance of male to female plants frequently departs from the expected 50/50 ratio due to ecological and/or demographic factors (Field, Pickup & Barrett, 2013). It is also known that in some dioecious plants with clonal reproduction, there is a tendency for the sex ratio to take longer to come into balance (Field, Pickup & Barrett, 2013). In small, isolated populations of El Dorado bedstraw that start with a skewed sex balance, it is possible that genetic diversity could be further eroded due to a lack of diverse mating opportunities. Furthermore, variation in climate across the small but topographically rugged Pine Hill region (Fig. 1) may have led to the evolution of associations between genetics and climate, further limiting the dispersal potential of plants, seeds, and pollen (Kubisch et al., 2013; Hargreaves, Bailey & Laird, 2015).

To implement conservation and recovery for this federally endangered taxon, there is a need for more information on genetic patterns and reproductive biology. Here, double-digest, restriction site-associated DNA (ddRAD) sequencing (Peterson et al., 2012) is used to develop a single nucleotide polymorphism (SNP) dataset for a large sample of El Dorado bedstraw (Fig. 1). Potential pollen dispersers are studied via observation of floral visitation. Genetic data are used to estimate population genetic parameters and determine (1) which populations are genetically isolated, (2) which populations are most genetically diverse and (3) which populations contain an imbalance of male versus female genets. Based on these analyses, recommendations are made as to which populations should receive focused conservation effort.

## Materials and Methods

### Sampling

For sampling of El Dorado bedstraw, seven locations in the Pine Hill area were selected (Table 1; Fig. 1). To prevent confusion between populations and subpopulations, plant collecting locations are referred to as “locales”. Locales are groupings of plants that are spatially discontinuous from other groupings of plants, with their centers at least 1,000 m apart and their edges at least 100 m apart. Sampling was carried out using a spatially explicit, repeatable transect approach. At each locale, a 50 m linear transect was installed. On each transect, 12 random numbers between zero and 50 were selected (with replacement), and a single ramet sampled at each position. When no plants were present at a selected position, the nearest ramet was sampled, no less than 10 cm from the nearest other sampled ramet . In cases where the same position was selected more than once, a different stem was selected for each sample, no more than 5 cm from the first stem. For all sampled stems, apical meristems, young leaves, and in some cases flower buds were collected. Tissue was preserved in absolute ethanol. The sex of each sampled stem was noted at the time of collection using a 40 X lens (Table S1). Collections were carried out on the Pine Hill Preserve, which is managed by the Motherlode Field Office the United States Bureau of Land Management. Permission to collect on these lands was granted by the Preserve manager, Graciela Hinshaw. Collections were carried out with G. Hinshaw under a United States Fish and Wildlife Service recovery permit (TE-062125-4) to G. Hinshaw.

In addition to the population samples of the target taxon, El Dorado bedstraw, a total of 13 other samples from closely related taxa were obtained from around the state (Table S1). These were obtained for later use in reference genome sampling and for inclusion in RAD sequencing for later use as potential outgroups.

### Reference genome

Due to the lack of a reference genome for any species of *Galium*, a reference genome for the genus was prepared to aid in genotyping. A sample of the diploid *G. porrigens* (D. O. Burge 2233; Table S1) was used to avoid well known problems associated with assembly of polyploids (Renny-Byfield & Wendel, 2014). *Galium porrigens* is closely related to the focal taxon (Soza & Olmstead, 2010a), so genomic synteny is expected to be strong between the two taxa, facilitating genotyping. DNA for *G. porrigens* was extracted by LGC Genomics (Berlin, Germany) using the LGC sbeadex maxi plant kit. Extraction was done according to the manufacturer’s instructions, with four exceptions: (1) the sample was lyophilized for one hour in a SpeedVac (ThermoFisher, Waltham, MA, USA) prior to disruption; (2) dried tissue was disrupted using a Geno/Grinder (SPEX SamplePrep, Metuchen, NJ, USA) for 2 min at 1,750×*g* ; (3) lysis was done with the addition of RNase A for 30 min at 65 °C; and (4) washing was done in three steps, with wash buffers PN1, PN1 and PN2 from the sbeadex maxi plant kit. The final amount of DNA obtained was ~1 μg.

DNA quality control, library construction, and sequencing were also conducted at LGC Genomics. Briefly, genomic DNA was fragmented in an ultrasonicator (Covaris, Chicago, IL, USA), with the target fragment size set to 300 bp. Fragmented DNA was purified in PEG. The resulting purified DNA was used to prepare the Illumina sequencing library using the Ovation Rapid DR Multiplex System 1-96 (NuGEN, San Carlos, CA, USA) including the following steps: End Repair, Ligation, Final Repair, Library Purification and Library Amplification. After amplification, the Illumina library was purified and size selected using gel electrophoresis. Quality control of the DNA library was done using a Bioanalyzer (Agilent, Santa Clara, CA, USA) and a Qubit 4 Fluorometer (ThermoFisher, Waltham, MA, USA). The library was sequenced on an Illumina NextSeq 500 in 150 bp, paired-end mode, with all of the samples multiplexed in the instrument’s single flow cell. As there are no estimates for genome size in *G. porrigens* or any close relatives, it was not possible to assess the likely coverage of this amount of sequencing against the *G. porrigens* genome.

Following sequencing, demultiplexing of the libraries was done using Illumina bcl2fastq software, version 1.8.4. Up to two mismatches or Ns were allowed in the barcode read. FASTX-Toolkit (http://hannonlab.cshl.edu/fastx_toolkit) was used to do the following quality control steps: (1) remove sequence adapter remnants and ddRAD cut-site sequences from all raw reads, (2) remove reads with a final length <20 bp, (3) remove reads containing more than one N and (4) trim reads at the 3′ end to get a minimum average Phred quality score of 10 over a window of 10 bases. Error correction of quality trimmed reads was done using Musket version 1.0.6 (http://musket.sourceforge.net/) with a *k*-mer size of 21.

Assembly of shotgun DNA sequences was done using the CLC Genomics Workbench, version 8.0 (Qiagen, Venlo, the Netherlands) under the following assembly parameters: mapping mode = map reads back to contigs; update contigs = yes; automatic bubble size = yes; minimum contig length = 200; word size = yes; perform scaffolding = yes; auto-detect paired distances = yes; mismatch cost = 2; insertion cost = 3; deletion cost = 3; length fraction = 0.5; similarity fraction = 0.8; create list of un-mapped reads = no.

### Double digest RAD sequencing

Genomic DNA for ddRAD sequencing was extracted by LGC Genomics as described above for the reference genome. DNA quality control, library construction, and sequencing were also done at LGC Genomics. Briefly, 100–200 ng of genomic DNA from each of 96 samples (Table S1) were digested with two units each of the restriction enzymes *MspI* and *PstI*-HF (New England Biolabs, Ipswich, MA, USA; hereafter referred to as NEB) using 1x CutSmart Buffer (NEB) in a 20 μl volume for 2 h at 37 °C. These enzymes were used due to ongoing research at LGC Genomics that indicated their efficacy in a broad set of plant species. The restriction enzymes were heat-inactivated by incubation at 80 °C for 20 min. Following restriction digestion, 10 μl of each restriction digest were mixed on ice with 1.5 μl of inline-barcoded forward PstI adaptors (pre-hybridized, 5 pM/μl), followed by addition of 20 μl of ligation master mix (15 μl NEB Quick ligation buffer, 0.4 μl NEB Quick Ligase, and 5 pM pre-hybridized reverse MspI adaptor). A separate inline barcode was used for each sample. Ligation reactions were incubated for 1 h at room temperature, followed by inactivation for 10 min at 65 °C. All reactions were diluted with 30 μl TE (10 mM Tris/HCl, 50 mM EDTA, pH 8.0). Ligation reactions were then mixed with 50 μl Agencourt AMPure XP magnetic beads (Beckman Coulter Life Sciences, Pasadena, CA, USA), incubated for 10 min at room temperature, and placed for 5 min on a magnet to collect the beads. The supernatant was discarded and the beads washed two times with 200 μl 80% ethanol. Beads were air dried for 10 min and libraries eluted in 20 μl Tris Buffer (five mM Tris/HCl, pH 9.0). 10 μl of each library were amplified in 20 μl PCR reactions using MyTaq (Bioline Reagents, London, UK) and Illumina TrueSeq primers. Cycle number was limited to 14.

A total of 5 μl from each of the amplified libraries were pooled. PCR primers and small amplicons were removed from this pooled sample by Agencourt XP bead purification using 1 volume of beads. PCR enzyme was removed from the pooled sample using a Qiagen MinElute Column. The pooled sample was eluted in a final volume of 20μl Tris Buffer (five mM Tris/HCl, pH 9.0). Normalization of the pooled library was done using the Evrogen TRIMMER kit (Moscow, Russia); 12 μl of the pooled ddRAD library (a total of 1 μg DNA) was mixed with 4 μl of the 4x hybridization buffer, denatured for 3 min at 98 °C, and incubated for 5 h at 68 °C to allow for reassociation of the DNA fragments. A total of 20 μl of the 2x DSN master buffer was added and the samples were incubated for 10 min at 68 °C. One Unit of the DSN enzyme (1 u/μl) was added and the reaction was incubated for another 30 min. The reaction was terminated by the addition of 20 μl DSN Stop Solution, then purified on a Qiagen MinElute Column and eluted in 10μl Tris Buffer (five mM Tris/HCl, pH 9.0). Following normalization, the library was re-amplified in a 100 μl PCR reaction using MyTaq. An i5-Adaptor primer was used in this reaction. Cycle number was limited to 14.

The ddRAD library was size selected on a BluePippin (Sage Science, Beverly, MA, USA), followed by a second size selection on a low melting point agarose gel. In both cases, the target was to removing fragments smaller than 200 bp and larger than 400 bp. The pool was sequenced on a single rune of an Illumina NextSeq 500 using V2 chemistry and 300 cycles (200 M reads).

### Variant detection and filtering

Following sequencing, DNA sequences from individual plants were separated according to their barcodes, and the barcodes trimmed. BWA (Li & Durbin, 2009) was used to align DNA sequences to the reference genome. Resulting BAM files were indexed using SAMTOOLS (Li et al., 2009). Alleles were called using the HaplotypeCaller function of GATK (McKenna et al., 2010), with ploidy set to eight. Each sample was then genotyped using the GenotypeGVCFs function of GATK. Variants were filtered using the VariantFiltration function in GATK, removing all variants with confidence by depth less than 2 (QD < 2.0) and mapping quality less than 20 (MQ < 20.0). QD is the quality score (QUAL) normalized by unfiltered depth, and accounts for samples with excessive coverage; MQ is the u-based z-approximation from the Mann-Whitney-Wilcoxon Rank Sum Test for mapping qualities (MAPQ of reads supporting REF vs. MAPQ of reads supporting ALT). The SelectVariants module of GATK was used to remove invariant sites (-env), exclude loci with one allele (--restrictAllelesTo MULTIALLELIC), restrict the dataset to SNPs (-selectType SNP), and remove loci with more than 20% missing data (--maxNOCALLnumber 20).

As there are no estimates for genome size in El Dorado bedstraw or any close relatives, it was not possible to assess the likely coverage of this amount of sequencing against the plant’s genome. In the absence of such coverage estimates, it is likewise not possible to assess how coverage might effect SNP calling and other down-stream methods applied to the data.

The dataset was not filtered against loci under selection. Although it would be preferable to exclude selected loci from the dataset in the interest of conducting subsequent analyses on data that is selectively neutral, there is a lack of existing software or methods that allow for such filtering in polyploid SNP datasets, as most such genome scanning tools rely on the assumption that the loci are diploid (Foll & Gaggiotti, 2008). Instead, I have assumed that the data consists of neutral loci, similar to the assumption made in most population genetic research on polyploids that makes use of microsatellites (Vieira et al., 2016).

### Measuring clonality

As is the case with many species of *Galium*, and especially *G. californicum*, El Dorado bedstraw is capable of spreading asexually via stem layering. As is the case with all organisms capable of clonal reproduction, this can lead to a single genet becoming represented by multiple ramets derived from the original genet. Clonality was quantified by identifying the number of unique multilocus genotypes (MLGs) and assessing whether these are the product of asexual reproduction. When an MLG is shared among multiple samples, this does not mean that the samples belong to one genet and are the product of asexual reproduction; the dataset may lack power to detect the genetic differences between closely related plants (e.g., siblings) that are derived from sexual reproduction. It is also possible that multiple MLGs belong to one genet, the genetic differences resulting from somatic mutation. In the latter case, the group is best referred to as a multilocus lineage (MLL). However, definition of MLLs relies on selection of a genetic distance threshold to group MLGs into MLLs (Bailleul et al., 2016). Some researchers have solved this problem by collecting genetic data on known ramets in order to directly quantify the amount of genetic differentiation expected among ramets (Douhovnikoff & Dodd, 2003). However, such studies are very rarely done due to the logistical hurdles involved. More frequently, workers have relied on the following categories of methodology to group MLGs: (1) clustering algorithms (Kamvar, Brooks & Grünwald, 2015), (2) modeling asexual versus sexual reproduction (Bailleul et al., 2016), (3) visual examination of frequency distributions of genetic distance (Meirmans & Van Tienderen, 2004; Arnaud-Haond & Belkhir, 2007; Clark & Jasieniuk, 2011) and (4) various combinations of these methods. In all such methods, however, the definition of MLLs depends, either directly or indirectly, on assumptions made by the user regarding appropriate genetic distance thresholds or population genetic parameters (e.g., the mutation rate). What is more, some of these methods lack implementations for polyploids.

Methods to quantify clonality in the present study were selected based on (1) their availability in a form applicable to polyploids and (2) the number of assumptions, especially with respect to population genetic or demographic parameters that were not directly measured. First, the R package *poppr* v. 2.8.3 (Kamvar, Tabima & Grünwald, 2014) was used to group MLGs into MLLs based on a-priori selection of a genetic distance threshold to define the MLLs. To test the sensitivity to the genetic distance method, the analysis was run using dissimilarity distance (*diss.dist* in *poppr*) and Nei’s distance (*nei.dist* in *poppr*). The *filter_stats* algorithm was applied under default parameters, identifying three alternative genetic distances thresholds that could be used to identify MLLs for each of the distance matrices. As described above, using a genetic distance threshold to distinguish one MLL from another allows for small differences among MLGs, such as those derived from DNA sequencing error or somatic mutations. For each genetic distance method, the *mlg.filter* option of *poppr* was used, under default settings, to collapse MLGs into MLLs based on each of three genetic distance thresholds, *farthest_neighbor* (merges MLGs based on maximum distance between individuals in either of the clusters; most strict), *average_neighbor* (merges MLGs based on average distance between every pair of individuals between clusters; intermediate strictness), and *nearest_neighbor* (merges MLGs based on minimum distance between individuals in either cluster; least strict). Each use of *mlg.filter* was implemented under default parameters.

As an alternative method of inferring the degree to which plants reproduce asexually, the index of association (*I*_*A*_; Smith et al., 1993) and the index of multilocus linkage disequilibrium ($\bar r$_*d*_; Agapow & Burt, 2001) were inferred for each locale. Calculations of these indices were done in *poppr* (Kamvar, Tabima & Grünwald, 2014). Both indices express linkage disequilibrium. As such, both tend to be higher in populations where there is a higher level of asexual reproduction. The *I*_*A*_ index is similar to $\bar r$_*d*_, but the latter accounts for the number of loci (Kamvar, Tabima & Grünwald, 2014).

### Population genetic statistics

Population genetic analysis was used to obtain genetic diversity statistics and estimate differentiation among locales. The program GENODIVE (Meirmans & Van Tienderen, 2004), along with the R packages *adegenet*, version 1.3-1 (Jombart, 2008; Jombart & Ahmed, 2011) and *poppr* (Kamvar, Tabima & Grünwald, 2014) were used to calculate population genetic statistics. GENODIVE was used to calculate both observed (*H*_O_) and expected (*H*_E_) heterozygosity, as this software aims to account for polyploidy in calculating these statistics. When applied to polyploids, GENODIVE uses gametic heterozygosity (Moody, Mueller & Soltis, 1993) for *H*_O_. Gametic heterozygosity is the chance that two random alleles drawn from an individual are the same (Moody, Mueller & Soltis, 1993). In GENODIVE, both estimates of heterozygosity are calculated taking into account the potential for allele dosage errors introduced by allele calling in a polyploid. However, such dosage errors should be minimized by GATK, which accounts for allele dosage during allele calling (McKenna et al., 2010).

The package *adegenet* was used to calculate allelic richness (sensu Greenbaum et al., 2014) for each locale, and to estimate differentiation (*F*_ST_) among the locales. Isolation by distance was assessed using partial Mantel tests in the R package *vegan* version 2.5-1 (Oksanen et al., 2018), controlling for location (latitude and longitude) of the locale. For the Mantel tests, both Pearson and Spearman correlation coefficients were used with 1,000 permutation test replicates.

### Relationships among populations

Tree-building was used out as a means to visually assess and test relationships among samples and locales. The R package *poppr* (Kamvar, Tabima & Grünwald, 2014) was used to build a neighbor-joining dendrogram. The *aboot* algorithm of *poppr* was applied to dissimilarity distance with 1,000 bootstrap replicates; all other parameters were set to their default.

Principal components analysis (PCA) was used as a non-parametric means to assess the relationships among samples and locales. PCA was implemented in the R package *ade4*, version 1.7-13 (Dray & Dufour, 2007) using the *dudi.pca* algorithm.

### Pollinators

The diversity of potential pollinators of El Dorado bedstraw was assessed based on direct observation of floral visitation of both male and female plants at all seven of the locales targeted for DNA sequencing (Table 1). Observations were made in spring of 2017 and 2018. A total of 29 h of observations were done; at sites that were visited more than once, an attempt was made to vary the time of day, from dawn to dusk. During the observations, multiple colonies of both sexes were observed, and insects seen to visit flowers were collected using forceps, a net, or an aspirator, depending on the size and behavior of the insect. Insects were collected directly into 95% ethanol. All observations were done by the author, L. Couper and J. Marszalek. The insects were identified to family by L. Kimsey (Bohart Museum of Entomology, U. C. Davis); voucher specimens of these insects are deposited at the Bohart Museum of Entomology.

The diversity and abundance of pollinators at each locale was compared to population genetic parameters, including expected heterozygosity, allelic richness and number of MLLs. The diversity of pollinators was expressed using both species richness and the Shannon index, calculated using the R package *vegan* version 2.5-1 (Oksanen et al., 2018). Species richness is simply a count of the number of species present, while Shannon index accounts for abundance of each species (Hill, 1973). Insect diversity metrics were compared to the three population genetic parameters using linear regression, carried out using the *ml* function of R (R Development Core Team, 2020). Sampling effort (hours spent collecting pollinators at each locale) was added to each model. All variables were centered, scaled and transformed into *Z* scores prior to analysis using the scale function off R (R Development Core Team, 2020). The contribution of the two diversity variables and sampling effort on the model were assessed using ANOVA (*anova* function of R; R Development Core Team, 2020).

## Results

### Reference genome

After filtering, a total of 515,563,790 reads (including 257,781,895 pairs) were available for the sample of *G. porrigens* used in the whole genome shotgun sequencing (D. Burge 2233; Table S1). The reads were assembled into 274,635 scaffolds (330,745 contigs; N50: 66,857 bp) with a total scaffold length of 434,739,045 bp (430 MB). Raw sequences used to generate the genome are on GenBank (SAMN09761870). Genome contigs are available online (DOI 10.5281/zenodo.3836756).

### Genotypes

A total of 84 plants from seven locales were subjected to ddRAD sequencing (Table S1). Quality was high for all but two samples, 2275-2 and 2275-10; these were excluded from genotyping due to low sequencing coverage and missing data. For the remaining 82 samples, coverage averaged 3,102,452 reads per individual (SD: 1,049,103 reads; Table S2). Raw DNA sequence data are available at GenBank (PRJNA484218). Following assembly, genotype calling and filtering, the dataset contained 20,255 loci. This dataset is available online as a VCF file (DOI 10.5281/zenodo.3836756).

### Population genetics

Allelic richness varied from 37,379 (locale 2281) to 40,564 alleles (locale 2260; Table 2). Observed heterozygosity (*H*_O_) varied from 0.083 (locale 2281) to 0.090 (locales 2260, 2263, 2272, and 2274; Table 2). Expected heterozygosity (*H*_E_) varied from 0.114 (locale 2281) to 0.118 (locales 2263, 2264, and 2274; Table 2). Genetic differentiation (*F*_ST_) varied from 0.04 (locale 2260 vs. 2272) to 0.06 (locale 2264 vs. 2281; Table S3). For tests of isolation by distance among locales, both Spearman (*P* = 0.0004; Mantel *r* statistic: 0.172) and Pearson methods (*P* = 0.001; Mantel *r* statistic: 0.242) yielded significant results.

**Table 2:** Population genetic and pollinator summary statistics.

	Locale name	*N*	Alleles	*H*_O_	*H*_E_	MLLs	Colonies	*I*_A_	$\bar r$_d_	Richness	Shannon
2260	Cameron Park	12	40,564	0.090	0.116	10	2	71.3	0.0062	10	1.47
2263	Tiffany Hill	12	38,990	0.090	0.118	6	3	235.9	0.0218	2	0.67
2264	Farview	12	38,203	0.087	0.118	6	5	156.1	0.0148	2	0.69
2272	Pine Hill	12	40,269	0.090	0.117	8	4	95.5	0.0084	6	0.95
2274	Lazy Knoll	12	39,894	0.090	0.118	8	3	139.7	0.0125	2	0.00
2275	Salmon Falls	10	37,381	0.088	0.117	5	4	185.2	0.0181	5	0.87
2281	Many Oaks	12	37,379	0.083	0.114	6	2	266.9	0.0264	1	0.00

**Note:**

N, refers to the number of individuals in the analysis; for locale 2,275, two sequenced individuals were discarded due to missing data. Alleles, refers to the number of alleles inferred for each locale, also known as allelic richness, calculated in the R package adegenet. *H*_O_, observed heterozygosity, calculated by GENODIVE (Meirmans & Van Tienderen, 2004) according to the method of (Moody, Mueller & Soltis, 1993), taking into account ambiguity in allele dosage due to polyploidy. *H*_E_, expected heterozygosity, calculated by GENODIVE (Meirmans & Van Tienderen, 2004), taking into account ambiguity in allele dosage due to polyploidy (Meirmans & Van Tienderen, 2004). MLLs, the number of multilocus lineages inferred by the R package poppr using dissimilarity distance. Colonies: number of MLLs represented by more than one individual (also referred to as clonal colonies or genets; also see Table 3). I_A_, index of association (Smith et al., 1993). <!--[if !msEquation]--> <!--[endif]-->_d_, Index of multilocus linkage disequilibrium (Agapow & Burt, 2001). Richness, number of pollinator species observed at the locale. Shannon, Shannon index of diversity (Hill, 1973).

**Table 3:** Clonal colonies detected. Based on the results of the R package poppr (Kamvar, Tabima & Grünwald, 2014). The mlg.filter option of poppr was used to collapse multilocus genotypes (MLGs) into multi-locus lineages (MLLs) based on dissimilarity distance (diss.dist in poppr). MLLs are equivalent to clonal colonies.

Locale	Sex	Ramets	Extent (m)	Samples (transect position, m)
2260	**♀**	2	0	2260-2 (14), 2260-3 (14)
	**♀**	2	0	2260-4 (15), 2260-5 (15)
2263	♂	3	4	2263-1 (4), 2263-2 (7), 2263-3 (8)
	**♀**	4	6	2263-4 (29), 2263-5 (30), 2263-6 (31), 2263-9 (35)
	♂	2	0	2263-7 (34), 2263-8 (34)
2264	**♀**	2	0	2264-1 (5), 2264-2 (5)
	**♀**	2	1	2264-3 (11), 2264-4 (12)
	♂	2	4	2264-5 (19), 2264-6 (23)
	♂	3	6	2264-7 (25), 2264-8 (26), 2264-9 (31)
	**♀**	2	0	2264-11 (42), 2264-12 (42)
2272	**♀**	2	7	2272-1 (6), 2272-4 (13)
	♀	2	4	2272-2 (8), 2272-3 (12)
	♀	2	0	2272-10 (36), 2272-9 (36)
	♀	2	4	2272-11 (45), 2272-12 (49)
2274	♂	2	1	2274-1 (4), 2274-2 (5)
	♀	3	2	2274-3 (22), 2274-4 (23), 2274-5 (24)
	♂	2	2	2274-8 (37), 2274-9 (39)
2275	♂	3	3	2275-1 (1), 2275-3 (3), 2275-4 (4)
	♂	2	5	2275-6 (31), 2275-7 (36)
	♂	2	1	2275-8 (37), 2275-9 (38)
	♀	2	1	2275-11 (44), 2275-12 (45)
2281	♀	6	9	2281-1 (9), 2281-2 (9), 2281-3 (11), 2281-4 (12), 2281-6 (17), 2281-7 (18)
	♀^1^	2	1	2281-11 (41), 2281-12 (42)

**Note:**

1 Sample 2281-11 is ♀; the sex of sample 2281-12 is unknown.

**Figure 2:**
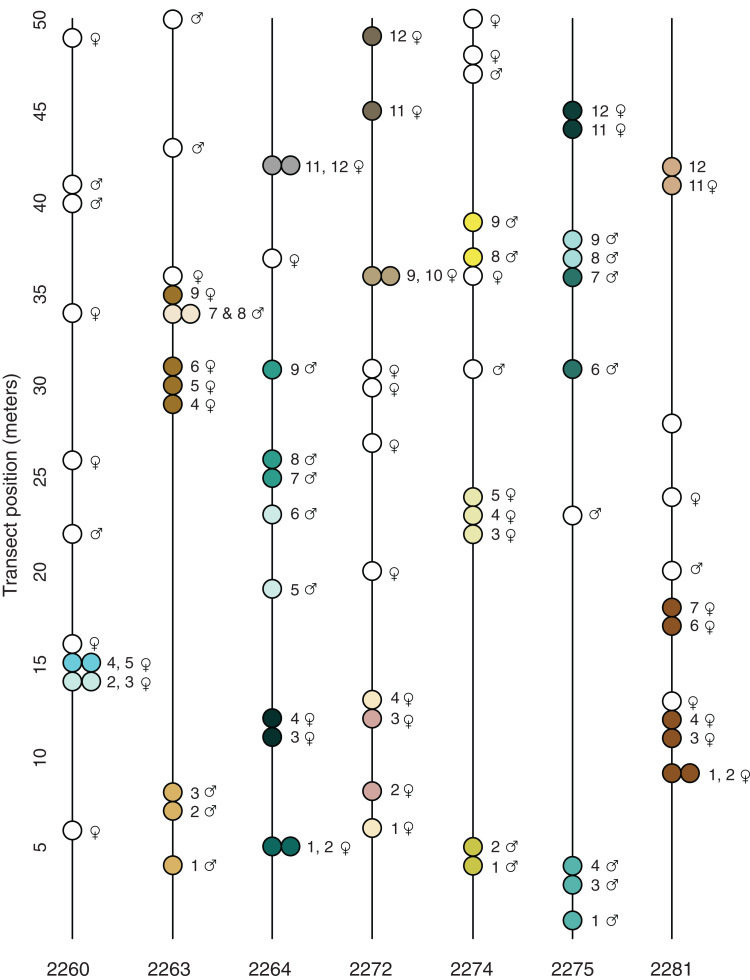
Transect position and clonal colony membership. Lines show the position of each sampled plant along the transect (direction does not reflect position of transect in the field); filled circles show which plants on a given transect belong to the same clonal colony (Table 3); empty circles are unique multi-locus lineages known from just one sampled stem. None of the detected MLLs were found in more than one locale.

Sex, the sex of the stems in the colony. Ramets, the number of stems that belong to a clonal colony. Extent, the maximum number of meters between stems from a colony. Samples, the names of the samples and their transect positions (the latter in parentheses). Also see Fig. 2.

### Clonality

Of the tested distance matrices, only dissimilarity distance collapsed MLGs into MLLs; when applied to the dissimilarity distance matrix, all three genetic distance thresholds collapsed the MLGs into the same 49 MLLs, with between 5 and 10 MLLs per locale (Table 2); none of the MLLs were shared between locales; 23 MLLs were represented by two or more individuals, and are considered to be clonal colonies (Fig. 2; Table 3). The index of association (*I*_A_, Smith et al., 1993) and index of multilocus linkage disequilibrium ($\bar r$_d_; Agapow & Burt, 2001) were highest at the same locales where a low number of MLLs were detected: 2263, 2264, 2275 and 2281 (Table 2).

The spatial distribution of the 23 potential clonal colonies (MLLs represented by more than one sample; Fig. 2) suggests that most clonal colonies are small and contained, the average spatial extent of such colonies being 2.7 m (Table 3). However, at the Many Oaks locale (2281; Table 3), a single MLL was sampled at up to 9 m horizontal distance (Fig. 2), which suggests that colonies may be able to cover extensive areas under certain conditions. In addition, some of the clonal colonies were not continuous (Table 3; Fig. 2), suggesting that these colonies may be larger than detected, or have given rise to satellite colonies established by asexual dispersal.

### Sex ratio

Most of the locales contained a relatively balanced number of female and male sampled stems, with a slight tendency toward females (average 7.0 ♀ and 4.7 ♂; Table 1). However, some of the locales were imbalanced, for example Pine Hill (locale 2272; 10 ♀ and two ♂) and Many Oaks (locale 2281; nine ♀, one ♂ and two unknown). What is more, the results of the tests for clonality suggest that female plants are better represented among the 23 detected clonal colonies (MLLs represented by more than one sample; Fig. 2; Table 3) than males, with 14 of the clonal colonies being female and nine male (including one MLL in which the sex of one of the individuals is not known; Table 3). The clonal colony with the largest number of samples is a female from the Many Oaks locale (2281; Table 1) with 6 sampled ramets spread over a distance of 9 m horizontal distance (Fig. 2).

### Neighbor-joining tree

**Figure 3:**
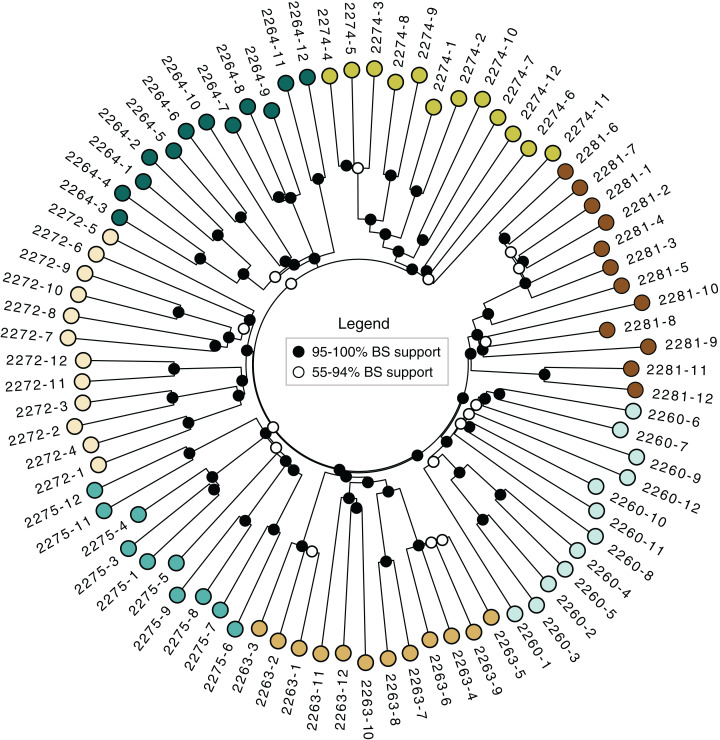
Neighbor-joining tree. Based on dissimilarity distances. Bootstrap support is from 1,000 bootstrap replicates. Branch lengths are proportional to dissimilarity distance.

The neighbor-joining tree (Fig. 3) revealed strong support for the distinctiveness of each locale, with higher than 95% bootstrap support for groups corresponding to each locale. Some of the locales also contained strong branching structure, suggesting a great deal of genetic structure even at the level of individual locales; all of the clonal colonies identified using the dissimilarity distance matrix in *poppr* (Table 3) have 95–100 % bootstrap support in the tree.

### Clustering

**Figure 4:**
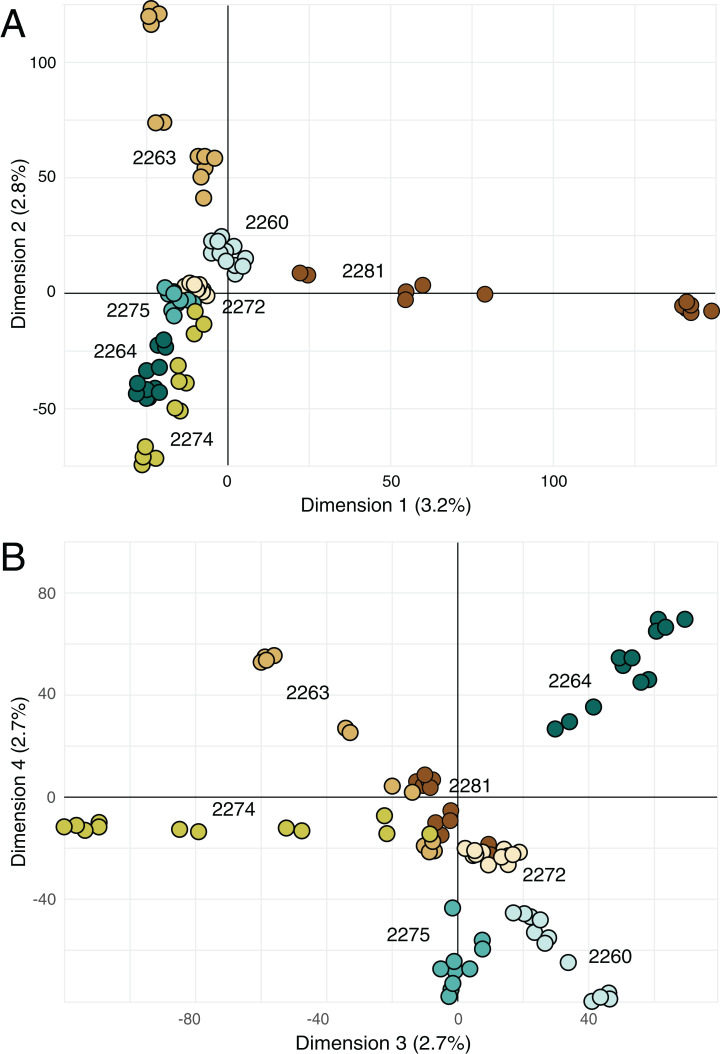
Principal components analysis on genetic data. (A) Biplot of first and second principal components derived from SNP data for all of the samples. (B) Biplot of third and fourth principal components derived from SNP data for all of the samples. Percentage reported on each axis is the amount of variance explained by each axis.

Principal components analysis (Fig. 4) highlighted the distinctiveness most locales across the four retained principal components; the single exception was locale 2272, which was not well differentiated from other locales on either combination of the retained axes. However, note that the retained principal components account for more only 11% of the variance in the data (Fig. 4).

### Pollinators

A total of 56 insects were collected from the flowers of El Dorado bedstraw during 29 h of observations over eight days in the months of May and June, 2017 and 2018 (Table 4). The insects were from three orders (Coleoptera, Diptera and Hymenoptera) and 11 families (Table 4). The most numerically dominant visitor among the Coleoptera (beetles) was a species of false flower beetle (Scraptiidae, 14 visits); among Diptera (flies), a species of midge (Cecidomyidae, nine visits); and among Hymenoptera (bees, ants and wasps), a species of ant (Formicidae, 16 visits).

**Table 4:** Pollinators collected from El Dorado bedstraw.

Code	Locale	Date	Hours	Order	Family	Number
2260	Cameron Park	12 May 2017	1	Coleoptera	Scraptiidae	1
		14 May 2017	2	Coleoptera	Elateridae	5
				Coleoptera	Scraptiidae	3
		22 May 2017	2	Diptera	Cecidomyidae	2
				Hymenoptera	Formicidae	2
				Coleoptera	Scraptiidae	5
		1 June 2018	4	Hymenoptera	Andrenidae	2
				Hymenoptera	Braconidae	1
				Hymenoptera	Formicidae	2
				Hymenoptera	Colletidae	1
2263	Tiffany Hill	22 May 2018	3	Diptera	Cecidomyidae	2
				Coleoptera	Scraptiidae	3
2264	Farview	31 May 2018	2	Diptera	Cecidomyidae	1
				Coleoptera	Pyrochroidae	1
2272	Pine Hill	18 May 2017	4	Hymenoptera	Braconidae	1
				Hymenoptera	Formicidae	6
				Coleoptera	Pyrochroidae	1
		23 May 2017	3	Hymenoptera	Eulophidae	1
				Hymenoptera	Formicidae	2
				Coleoptera	Pyrochroidae	2
2274	Lazy Knoll	13 May 2017	2	Hymenoptera	Formicidae	1
				Coleoptera	Melandryidae	1
2275	Salmon Falls	12 May 2017	3	Diptera	Cecidomyidae	4
				Hymenoptera	Formicidae	1
				Diptera	Syrphidae	1
		30 May 2018	2	Hymenoptera	Formicidae	2
				Coleoptera	Scraptiidae	1
2281	Many Oaks	31 May 2018	1	Coleoptera	Scraptiidae	1

**Note:**

Date, date of the observation; Hours, number of hours spent at locale on that date; Order, insect order of flower visitors; Family, insect family of flower visitors; Number, number of this type collected.

Pollinator species richness and diversity was highest at Locale 2260 (Cameron Park; Table 2). It was lowest at 2274 (Lazy Knoll) and 2281 (Many Oaks), although this may be an artifact of the low sampling effort at these two locales. Multiple linear regression of MLL against Shannon index, insect species richness, and sampling effort did not yield a significant model (*P* = 0.34; *R* = 0.25, D.F = 3, *F* = 1.67); this was also the case using expected heterozygosity (*P* = 0.81; *R* = −0.51, D.F = 3, *F* = 0.32) and allelic richness (*P* = 0.52; *R* = −0.03, D.F = 3, *F* = 0.94). ANOVA suggested that none of the predictor variables were significant in any of the models at the *P* < 0.05 level.

## Discussion

### Population differentiation

The sampled locales represent all of the known population centers of the focal taxon, and span the entire range of soil types and climatic conditions under which the plant grows in its very limited (less than 50 km^2^) range. Significant isolation by distance was detected across the study area using Mantel tests, suggesting that spatial distance is a major driver of genetic isolation. On the other hand, the results of the neighbor-joining tree (Fig. 3) and clustering results (Fig. 4) suggest that there is population differentiation at a relatively small spatial scale that is probably not entirely due to isolation by distance. For example, the neighbor joining tree (Fig. 3) yielded strong support for the genetic cohesiveness of each of the locales, despite the fact that some of them are very close to one another, for example 2264, 2272 and 2274, which are less than 1,200 m from one another (Fig. 1).

Contrary to the isolation by distance and neighbor-joining tree results, *F*_ST_ values (Table S3) suggest that there is low differentiation among the locales. As reviewed by Meirmans, Liu & Van Tienderen (2018), under the same population model, *F*_ST_ is always expected to be lower in a polyploid versus a diploid due to (1) more mutation events in a polyploid population as a result of the larger number of genomes, (2) a higher impact of migration due to the higher number of genomes arriving with each migrant and (3) weaker genetic drift. This theoretical expectation is borne out by empirical studies that compare polyploids to closely related diploids, either within a species or within a genus (Samuel, Pinsker & Ehrendorfer, 1990; Kloda et al., 2008; Kim, Shin & Choi, 2009; Kolář et al., 2012). In the absence of genetic data for a close diploid relative of El Dorado bedstraw (e.g., *G. californicum* subsp. *primum*, the only known diploid member of *G. californicum*), it is difficult to assess whether the low *F*_ST_ values seen here are lower than one might expect. Overall, it is not clear what may have given rise to the low level of genetic differentiation suggested by *F*_ST_.

Future population genetic studies on El Dorado bedstraw would benefit from additional DNA sequencing and analysis to ascertain allo-or autoploid origin and mode of inheritance (e.g., polysomic, disomic, or a mixture of the two; Bourke et al., 2018). Phylogenetic analysis of the kind used by Roux & Pannell (2015) could be applied to determine whether the taxon is of allo-vs. autoploid origin. However, this would require strongly supported phylogenetic information that places the focal taxon in the context of its close relatives, especially diploids; at the moment, no such information is available, and is beyond the scope of the present study. In terms of mode of inheritance, additional DNA sequencing and coalescent analysis of the kind used by St. Onge et al. (2012) could be used to establish the mode of inheritance in El Dorado bedstraw. With these two pieces of information in hand, it would be possible to test for natural selection at individual loci, which would allow for population genetic parameters, including *F*_ST_ as well as downstream parameters like clonality, to be calculated using only neutral loci.

### Asexual reproduction

Prior to the present study, nothing was known about reproduction in El Dorado bedstraw other than the fact that the taxon was dioecious (Soza, 2012) and had low seed set in most years (G. Hinshaw, 2019, personal communication). This, combined with the observation that the plant sometimes covers large areas with a dense growth of stems led to the assumption that asexual reproduction via stem layering might be a significant contributor to reproduction. Results presented here seem to suggest that this assumption might be correct; of the 82 stems that were successfully sequenced, 49 multilocus lineages were identified, with 23 of these made up of multiple samples, and thus inferred to be clonal colonies (Fig. 2; Tables 2 and 3). These 23 clonal colonies contain 56 of the stems (average 2.4 stems), which implies that on average, around 40% of the stems in a typical population are derived from asexual reproduction.

The clonal colonies appear to be predominantly female (61%; Table 3) even though the sampling revealed a relatively even balance of male and female stems across the sampled locales (Table 1; Fig. 2; Table S1). The predominance of female clonal colonies may indicate that this sex is more successful in clonal reproduction than male plants, which could have implications for the population genetics of the taxon (Barrett, 2015); if sex ratios are skewed by asymmetrical success of female colonies, populations could end up with insufficient numbers of male plants for sexual reproduction, with consequences for both fertility and mating. Indeed, locale 2281 appears to be in danger of such a process, as this is a very small and isolated patch (less than ~500 m^2^) that is apparently dominated by a single large female colony (Table 3; Fig. 2).

The results presented here also suggest that some clonal colonies have successfully spread over large areas, up to 9 m horizontal distance (Fig. 2; Table 3). Based on the growth habit of El Dorado bedstraw, which has slender, fragile stems that root at the nodes, such spread is either via episodic dispersal of stem fragments, or via slow, creeping spread of stems as they grow outward from the original plant. As outlined by Barrett (2015), asexual dispersal has the advantage of exposing a genet to sexual opportunities that it might not have at its original location. However, dispersal also has the potential to exacerbate loss of genetic diversity in the population by allowing particular genets to persist at the expense of sexual recruitment.

Although not all of the results presented here support a high rate of asexual reproduction, the independently calculated index of association and index of multilocus linkage disequilibrium (Table 2) agree with the number of clonal colonies inferred for each locale using the dissimilarity distance matrix in *poppr* (Table 3); the indices are highest in the same locales where the largest number of clonal colonies were identified. Despite the agreement of the population genetic indices with the dissimilarity distance results from *poppr*, it is possible that at least some of the MLG identified as products of asexual reproduction by *poppr* are actually the product of sexual reproduction in locales with extremely low levels of segregating variation. The low vagility of most pollinators identified in the present study (Table 4), combined with the dioecious sexual system of the plants, imply that little pollen is dispersed among locales, a condition that would tend to limit the influx of genetic variation into locales (Barrett, 1998). The lower frequency of male stems seen across the locales (Table 1) might also impede the spread of genetic variation due to pollen supply limitation (Barrett, 1998). Finally, the octoploid genome of the focal taxon could slow the spread of genetic variation within and among populations, as the four-fold higher number of genomes (compared to a diploid) present in a population should increase the time needed for genetic variation to proliferate (Monnahan & Brandvain, 2020).

Results presented here suggest that the amount of asexual reproduction varies strongly across locales (Tables 2 and 3). However, the present research does not directly address predictors of clonality. Finer-scale population genetic analysis, for example on continuous patches below 1 m horizontal distance, and in two spatial dimensions, would help to place a more precise number on the scale at which clonality occurs, and the manner in which clones spread.

### Conservation implications

Conservation and recovery of rare plants requires knowledge of population genetic patterns (Allendorf, Hohenlohe & Luikart, 2010). Genetic data can be used to (1) prioritize acquisition of lands that support unique genetic subunits of the taxon (Petit, Mousadik & Pons, 1998; Diniz-Filho et al., 2012), (2) set the stringency of protection for populations depending on genetic patterns (Petit, Mousadik & Pons, 1998; Diniz-Filho et al., 2012) and (3) select germplasm for ex-situ conservation or re-population programs (Guerrant, Havens & Vitt, 2014; Hoban & Schlarbaum, 2014; Mijangos et al., 2015; Gargiulo et al., 2019).

Land acquisition to support conservation of El Dorado bedstraw should prioritize locales that are known to contain unique subsets of genetic diversity, or are located in parts of the Pine Hill area where unique subsets of diversity has been identified, for example, in and around the Tiffany Hill locale (2263; Table 1). However, without prior DNA sequencing of plants from lands being considered for acquisition, it would be impossible to infer whether such plants represent unique subsets of variation not present at other locations.

In terms of setting the stringency for protection of plants in presently conserved lands, the Many Oaks locale (2281; Fig. 1) clearly deserves high priority. This is due to the evidence for a high rate of asexual reproduction, with more than half of the sampled stems belonging to a single MLL (Table 2). The high rate of clonal reproduction may be a consequence of the small number of plants, which occupy an area of only about 500 m^2^ (G. Hinshaw, 2017, personal communication). In such a small population, asexual reproduction could overwhelm sexual reproduction (Holsinger, 2000; Barrett, 1998, 2015). As discussed above, the dioecious sexual system of El Dorado bedstraw could exacerbate such a process if one sex has a higher rate of asexual reproduction, as appears to be the case at Many Oaks, which is dominated by one female clonal colony (Table 3). In such a location, more stringent protections, for example, limiting public access, could help to prevent population perturbations that might lead to further losses of diversity.

In addition to targeting at-risk locales like Many Oaks (2281; Table 1), as well as ones supporting unique subsets of variation, like Tiffany Hill (2263; Table 1), a genetically-informed conservation strategy on existing conserved lands should ensure the persistence of a set of populations that represent the complete suite of genetic diversity discovered by this study. In the case of El Dorado bedstraw, every sampled locale is genetically distinctive (Fig. 3), making it difficult to select a priority subset. However, some locales support higher genetic diversity than others (e.g., Cameron Park, locale 2260; Table 2), and should therefore be prioritized, so as to economize limited conservation resources.

If ex-situ conservation or seed banking (Guerrant, Havens & Vitt, 2014; Hoban & Schlarbaum, 2014; Mijangos et al., 2015) is carried out in El Dorado bedstraw, the program should include as many locales as possible, setting the priority for the locales based on genetic distinctiveness and the proportion of taxon-wide variation represented. For example, locales 2281 (Many Oaks) and 2263 (Tiffany Hill; Table 1) should be included on the basis of their unique subset of genetic variation and vulnerability to loss of genetic diversity due to asexual reproduction (respectively), while locales with high genetic diversity (e.g., Cameron Park, 2260) should be added to ensure a high proportion of genetic variation in the germplasm. If seed banking is done, an effort should be made to ensure that seeds are collected in a fashion that accounts for clonality. For example, sampling could be carried out with a minimum distance among sampled stems of 2.6 m, which is the average horizontal scale that clonal colonies occupied in this study (Table 3).

Overall, the apparent high rate of clonality detected in the present study (Tables 2 and 3) has clear conservation implications for El Dorado bedstraw, as the amount of asexual reproduction relative to sexual reproduction influences the genetic variability (Table 2), which determines the speed at which populations adapt (Barrett, 1998, 2015); highly clonal populations may not contain enough genetic variation to respond to future environmental changes like climate change, habitat fragmentation, and invasive species (Loehle, 1987; Eckert, 2001). This is a taxon-wide issue that engenders unique conservation needs and considerations going into an uncertain future.

## Conclusions

The high rate of clonality detected in El Dorado bedstraw, taken together with the sexual imbalance detected in some locales and the low vagility of the pollinators implies that the taxon is probably sensitive to population perturbations, and might be slow to adapt to changes in the environment. It is therefore recommended that conservation and recovery efforts take asexual reproduction, sex balance and pollination biology into account in land acquisition, recovery of small populations, ex-situ conservation and seed banking.

Future research should focus on pollen transfer, sexual recruitment, and small-scale population genetic patterns. Additional information on all of these topics will allow for broader generalizations on the population genetics of this plant, and allow for more specific and detailed recommendations for conservation. For example, data on pollen transfer and sexual recruitment would lead to the development of management tools, for example manual pollination, that could be used to maintain genetic diversity in this extremely rare plant.

## Supplemental Information

10.7717/peerj.10042/supp-1 Supplemental Information 1 Extended data for sampled El Dorado bedstraw. Data on samples of *Galium*, including (where relevant) sample code, locale name, transect position (m from start of transect), latitude, longitude, sex, and date collected. Latitude and longitude are in the WGS84 datum.

10.7717/peerj.10042/supp-2 Supplemental Information 2 DNA sequencing data. Sequencing coverage data and GenBank accession numbers for all 82 samples of El Dorado bedstraw used in population genetic analyses. Excludes two plants removed due to excessive missing data (2275-2 and 2275-10); these were not used in any of the analyses.

10.7717/peerj.10042/supp-3 Supplemental Information 3 Genetic differentiation (*F_st_*) among all seven locales. Calculated using the R package *adegenet*, version 1.3-1 (Jombart & Ahmed, 2011).

## Additional Information and Declarations

### Field Study Permissions

The following information was supplied relating to field study approvals (i.e., approving body and any reference numbers): Field collections were carried out on land belonging to the United States Bureau of Land Management (BLM), with permission from the managers of these lands under United States Fish and Wildlife Service recovery permit TE-062125-4. Permission to collect on these lands was granted by the preserve manager, Graciela Hinshaw.

### DNA Deposition

The following information was supplied regarding the deposition of DNA sequences: The raw DNA sequences from next-generation DNA sequencing are available at GenBank (PRJNA484218).

### Data Availability

The following information was supplied regarding data availability: The raw data is available at Dylan Burge. (2020). Conservation genomics and pollination biology of an endangered, edaphic-endemic, octoploid herb: El Dorado bedstraw (*Galium californicum* subsp. sierrae; Rubiaceae) (Data set). Zenodo. DOI 10.5281/zenodo.3836756
